# Is a knife the same as a plunger? Comparing the attentional effects of weapons and non-threatening unusual objects in dynamic scenes

**DOI:** 10.1186/s41235-024-00579-1

**Published:** 2024-10-08

**Authors:** Hannes M. Körner, Franz Faul, Antje Nuthmann

**Affiliations:** Institute of Psychology, Kiel University, Neufeldtstr. 4a, 24118 Kiel, Germany

**Keywords:** Weapon-focus effect, Attention, Eye movements, Eyewitness memory, Dynamic scenes

## Abstract

Observers’ memory for a person’s appearance can be compromised by the presence of a weapon, a phenomenon known as the weapon-focus effect (WFE). According to the unusual-item hypothesis, attention shifts from the perpetrator to the weapon because a weapon is an unusual object in many contexts. To test this assumption, we monitored participants’ eye movements while they watched a mock-crime video. The video was presented with sound and featured a female perpetrator holding either a weapon, a non-threatening unusual object, or a neutral object. Contrary to the predictions of current theories, there were no significant differences in total viewing times for the three objects. For the perpetrator, total viewing time was reduced when she held the non-threatening unusual object, but not when she held the weapon. However, weapon presence led to an attentional shift from the perpetrator’s face toward her body. Detailed time-course analyses revealed that the effects of object type were more pronounced during early scene viewing. Thus, our results do not support the idea of extended attentional shifts from the perpetrator toward the unusual objects, but instead suggest more complex attentional effects. Contrary to previous research, memory for the perpetrator’s appearance was not affected by object type. Thus, there was no WFE. An additional online experiment using the same videos and methodology produced a WFE, but this effect disappeared when the videos were presented without sound.

## Introduction

The weapon-focus effect (WFE) describes a phenomenon in which eyewitnesses to a crime recall the appearance of the perpetrator less accurately if the perpetrator was carrying a weapon. Currently, the theory with the most empirical support posits that the WFE occurs because weapons are unusual objects in many contexts (e.g., E. F. Loftus et al., 1987; Pickel, 2015). Objects which are not threatening in nature (i.e., not weapons) have been found to impair memory to a similar degree if they are also unusual in the given context (e.g., Mitchell et al., 1998; Pickel, 1998, 1999). Both weapons and non-threatening unusual objects are assumed to draw attention away from the perpetrator. Although this attention component of the WFE is an integral part of current theories, it has rarely been directly investigated. Results from our recent eye-tracking study on this topic (Körner et al., 2023) suggest that the attentional effects of weapons are actually more complex than previously assumed. The primary aim of the present study was to compare the attentional effects of weapons with those of non-threatening unusual objects using eye tracking and naturalistic dynamic stimuli (i.e., videos). Recall for the perpetrator’s appearance was also assessed.

E. F. Loftus et al. (1987) formulated two hypotheses to explain the WFE. The arousal/threat hypothesis proposes that the WFE occurs due to the threat posed by weapons. Specifically, weapons are assumed to heighten the level of arousal or stress of eyewitnesses. Based on Easterbrook’s (1959) cue-utilization hypothesis, this elevated arousal is assumed to in turn narrow eyewitnesses’ focus of attention, with witnesses focusing extensively on the weapon at the cost of attention paid to other aspects of the scene such as the perpetrator’s appearance.

In contrast, the unusual-item hypothesis attributes the WFE to the unusualness or unexpectedness of weapons in many contexts. Unusualness here refers to an incongruency with the spatial context (i.e., the scene). The unusual-item hypothesis was inspired by findings by G. R. Loftus and Mackworth (1978). In this widely cited study, participants viewed a set of line drawings for 4 s each. The authors manipulated the contextual congruency of a critical object. For example, a scene of a farm could contain either a tractor (context-congruent) or an octopus (context-incongruent). Results showed that incongruent objects were fixated earlier, more often, and for longer durations than congruent objects. Based on these findings by G. R. Loftus and Mackworth (1978), E. F. Loftus et al. (1987) suggested that the WFE may occur because eyewitnesses do not expect to see a weapon in a context in which it is unusual.

Since then, there has been a whole body of literature devoted to studying the attentional effects of context-incongruent objects (for a review, see Wu et al., 2014). Several studies replicated the finding that incongruent objects are fixated more often and for longer durations compared with congruent objects (e.g., Coco et al., 2020; Henderson et al., 1999; Võ & Henderson, 2009). Whether incongruent objects are also fixated earlier is less clear, with some studies finding such an effect (e.g., Coco et al., 2020; Underwood et al., 2008), whereas others found no differences between congruent and incongruent objects (e.g., Henderson et al., 1999; Võ & Henderson, 2009).

There are some methodological aspects of this basic scene-perception research that limit the generalization to the WFE. For one, all of these studies used static images such as line drawings (e.g., G. R. Loftus & Mackworth, 1978), computer renderings (e.g., Võ & Henderson, 2009), or photographs of real-world scenes (e.g., Coco et al., 2020). Dorr et al. (2010) compared participants’ eye movements while they watched natural scenes in the form of either videos or stop-motion sequences (i.e., slide shows, in which still images sampled from the respective video were presented for a few seconds each). The patterns of results depended on the mode of presentation, demonstrating that findings for (series of) static scenes do not necessarily generalize to dynamic scenes. Secondly, research on incongruent objects has used only comparatively short exposure durations (ranging from 4 to 15 s) and has focused primarily on measures of extrafoveal and first-pass foveal processing (i.e., the first time that objects are fixated). It is therefore not entirely clear to what extent the effects of context-congruency persist over time. However, there is some evidence that an attentional bias toward incongruent objects can be observed in overall gaze behavior during scene viewing for up to 15 s (e.g., Henderson et al., 1999).

In the context of the WFE, research investigating the role of unusualness has focused almost exclusively on memory effects. For example, it has been found that unusual objects like a whole raw chicken (Pickel, 1998) or a stalk of celery (Mitchell et al., 1998) lead to similar memory impairments as weapons do. Moreover, the extent to which weapons reduce memory accuracy seems to depend on how unusual they are in the given context. For example, weapons have been shown not to impair memory when they are congruent to the surrounding scene (e.g., a gun at a shooting range; Pickel, 1999). Similarly, weapon presence has been found not to reduce memory accuracy when the person carrying the weapon can be expected to be armed (e.g., a police officer; Pickel, 1999).

Conversely, memory impairments tend to be greater when the person carrying the weapon has characteristics that are less associated with weapons. Pickel (2009) found that the memory-related WFE was stronger for a female perpetrator than for a male perpetrator. By contrast, a knitting needle caused a greater memory impairment than a knife for a male perpetrator, whereas the opposite was true for a female perpetrator. The author attributed these results to gender stereotypes influencing the associations of certain objects with different gender roles and, consequently, the perceived unusualness of the given objects. Similar effects have since been reported for racial stereotypes (Pickel & Sneyd, 2018). Meta-analyses on the WFE (Fawcett et al., 2013; Kocab & Sporer, 2016) confirmed that non-threatening unusual objects impair memory to a similar degree as weapons do.

While these findings support the hypothesis that the unusualness of weapons in many contexts is a key reason why weapons impair memory accuracy, there is some evidence that threat also plays a role. For example, Kim et al. (2014) varied both threat and unusualness and found that both factors impaired memory independently of each other. Similarly, some studies have reported larger memory effects for weapons, which are both unusual and threatening, than for non-threatening unusual objects (Hope & Wright, 2007; Mansour et al., 2019). Meta-analyses have found the WFE to be stronger when the weapon is used in a threatening manner (Fawcett et al., 2013) or in the context of a crime (Kocab & Sporer, 2016).

In the broader literature on the effects of stress on eyewitness memory, stress has been shown to negatively impact memory in studies that successfully induced stress (Deffenbacher et al., 2004). It is important to note that ethical constraints limit the degree of arousal that can be induced in experimental research. It is possible that the effects of threat are exacerbated under the presumably extreme levels of stress experienced by eyewitnesses to real crimes, especially by victims. Since the arousal/threat hypothesis and the unusual-item hypothesis are not mutually exclusive, both threat and unusualness may contribute to the WFE.

In contrast to the memory effects of weapons, their attentional effects are less well understood. So far, the commonly accepted explanation has been that weapons capture eyewitnesses’ attention, and that this attentional focus leads to a poorer encoding of the perpetrator’s appearance. For example, Pickel (2015) defined the WFE as follows: “The *weapon-focus effect* is that witnesses tend to direct their attention toward a weapon held by a perpetrator, which causes them to remember the perpetrator’s physical features and clothing less accurately than they would have without the weapon’s presence” (p. 490). However, this attention component of the WFE has been studied far less extensively than the memory component.

The most direct evidence supporting the assumption that eyewitnesses focus their attention on the weapon stems from an eye-tracking study by ﻿E. F. Loftus et al. (1987). In this study, participants watched a slide show depicting a staged interaction between a man and a cashier at a restaurant. Depending on the experimental condition, the man held either a gun or a check. Eye-tracking results revealed that participants fixated the gun more often and for longer durations compared with the check. A subsequent study by Biggs et al. (2013) investigated the attentional effects of weapons using a different paradigm. Here, the authors presented participants with individual, non-narrative images each depicting a single person holding either a weapon or a neutral object. Results showed that participants spent more time looking at the weapons than at the neutral objects, thereby corroborating the findings of ﻿E. F. Loftus et al. (1987). Importantly, this increased viewing time on the weapons came at the cost of viewing time on the faces of the people holding them. Thus, these results provide evidence for an attentional shift from the person holding a weapon to the weapon itself (﻿E. F. Loftus et al., 1987, did not report fixations on the depicted people).

There is also evidence that non-threatening unusual objects bind attention to a similar degree as weapons do. Similar to ﻿E. F. Loftus et al. (1987), Hope and Wright (2007) presented participants with a slide show of a staged robbery. For the slide involving the critical object, participants were slower to react to a secondary task when the scene included a weapon or an unusual object than when it included a neutral object. In a study by Flowe et al. (2013), participants were instructed to look either toward or away from a target object, which appeared in the periphery and without a broader context. The authors recorded participants eye movements and used the saccade reaction time to measure the degree to which the objects engaged participants’ attention. Results showed that an uncommon object engaged attention to a greater extent than a neutral object. Results for the weapon were mixed, with the weapon attracting more attention than the neutral object in only one of the two experiments.

All of these studies on the attentional effects of weapons used static stimuli, in the form of either slide shows (Hope & Wright, 2007; ﻿E. F. Loftus et al., 1987) or non-narrative images (Biggs et al., 2013; Flowe et al., 2013). As noted above, attention allocation can be different for static stimuli compared with more naturalistic viewing conditions such as videos (Dorr et al., 2010). Relatedly, viewing behavior for highly-edited dynamic stimuli such as motion pictures has been shown to be strongly shaped by bottom-up factors, which seem to override many top-down influences typically observed for static stimuli (e.g., Hutson et al., 2017; Loschky et al., 2015). This strong influence of bottom-up factors leads to a high inter-observer attentional synchrony, a phenomenon Loschky et al. (2015) coined the tyranny of film. Recent findings suggest that the high attentional synchrony observed for movies is largely due to moving elements capturing the observers’ attention (Hutson et al., 2022; also see Itti, 2005). However, there is research suggesting that the tyranny of film is less pronounced for other types of dynamic scenes such as unedited real-world scenes (Dorr et al., 2010) or screen-captured instructional videos (Levin et al., 2021).

In our recent study (Körner et al., 2023), we used eye tracking to investigate whether weapons also attract attention in video stimuli. Contrary to previous findings, participants did not spend significantly more time looking at a gun than at a cell phone. Viewing time on the perpetrator was also not affected by the type of object he held. Moreover, both the weapon and the neutral object were looked at for much shorter durations compared with the depicted people. Instead of a simple attentional shift away from the perpetrator and toward the weapon, weapon presence seems to have led participants to focus more closely on the interaction of the depicted people.

To explore whether these unexpected results were due to the use of video stimuli, our previous study included a second eye-tracking experiment in which we converted the videos to slide shows, mimicking the methodology of ﻿E. F. Loftus et al. (1987). For these slide shows, we were able to replicate the attentional bias toward a weapon reported by ﻿E. F. Loftus et al. (1987). However, this increased focus on the weapon did not come at a cost in viewing time on the perpetrator, and viewing time on the weapon was still quite low overall. Finally, weapon presence did not impair memory in either eye-tracking experiment, nor in an additional online study with a larger sample size.

The current study expands upon these previous findings on the attentional effects of weapons. In our previous study (Körner et al., 2023), we compared a weapon to a neutral control object only. In such a design, it remains unclear whether any effects the weapon may induce are a result of its threatening nature or its unusualness in the given context. A central aim of the current study was to directly test these competing explanations by additionally including a non-threatening unusual object.

The stimuli used in Körner et al. (2023) were based on material from a recent US study (Pickel & Sneyd, 2018). The fact that Pickel and Sneyd (2018) found large memory effects for these videos allowed us to conclude that the absence of a WFE was unlikely to be due to specific stimulus characteristics. However, adapting the material from another study also had disadvantages. Most notably, the English soundtrack did not match the first language of our German participants. To account for this mismatch, we chose to omit the soundtrack in our previous eye-tracking experiments. For the current study, we created our own material, in which the protagonists spoke German.

Due to our unexpected, but consistent previous failures to replicate the memory-related WFE, we also designed the stimuli to elicit a maximum effect by choosing optimal values for known moderator variables. These moderator variables include (a) the gender of the perpetrator, (b) the weapon type, (c) the exposure duration of the weapon and the perpetrator, and (d) the demeanor of the perpetrator. Finally, we directly investigated the effects that including versus omitting the soundtrack has on the memory-related WFE (Experiment 2).

## Experiment 1

Participants watched a video of a staged robbery, while their eye movements were recorded. There were three versions of the video, which differed only with regard to the critical object held by the perpetrator. Depending on the experimental condition, the perpetrator carried either a weapon, a non-threatening and context-congruent object, or a non-threatening unusual object. After watching the video, participants completed a questionnaire about the appearance of the perpetrator.

If the unusualness of weapons is indeed a key factor underlying the WFE, then weapons and non-threatening objects should have similar effects on both attention and memory. If, on the other hand, effects differ for weapons and unusual objects, this would suggest that other characteristics of weapons, such as their threatening nature (cf. Easterbrook, 1959; ﻿E. F. Loftus et al., 1987), are more relevant for the WFE.

## Methods

### Design and participants

Object type (knife, water bottle, and plunger) was manipulated between-subjects. The sample consisted of *N* = 162 participants (123 women, 39 men) between 18 and 53 years of age (*M* = 24.0 years, *SD* = 5.6). Most participants were undergraduate psychology students. Subjects received either monetary compensation or course credit for their participation.

### Stimuli

The stimuli were designed to elicit a maximum effect based on known moderator variables of the (memory-related) WFE. One such moderator variable is the gender of the perpetrator. The memory-related WFE has been shown to be stronger for a female than for a male perpetrator (Pickel, 2009). Therefore, and firstly, the perpetrator in our videos was portrayed by a woman. Secondly, a knife was used as the weapon. In their meta-analysis, Kocab and Sporer (2016) found that studies using weapons other than guns (mostly knives, but also other objects such as a meat cleaver or a screwdriver) reported larger effects than those using guns. Kocab and Sporer (2016) had originally expected to find the opposite effect (i.e., a larger WFE for guns than for other weapons). To explain their findings, the authors hypothesized that because knives potentially cause more gruesome injuries than guns, knives may appear more frightening to the observer. Thirdly, both the perpetrator and the critical object were in view for a duration of approximately 12 s. A meta-analysis by Fawcett et al. (2013) has shown that the memory effects of weapons were strongest for exposure durations between 10 and 60 s. The main reason why we chose an exposure duration close to the lower end of this apparently optimal range was that in our previous study (Körner et al., 2023), the critical objects attracted attention primarily at the beginning of the scene. If there are differences between viewing times on the different objects, they are presumably most pronounced during this initial period. For shorter exposure durations, such initial differences have a greater impact on the overall effect, as the first few seconds make up a larger proportion of the total scene duration. Thus, the attentional WFE should be strongest for comparatively short exposure durations. Moreover, a recent study by Erickson et al. (2024) found that weapon presence impaired the recognizability of facial composites only at an exposure duration of 10 s, with no WFE observed at 30 s. Finally, the perpetrator in our videos used the weapon in a threatening manner, which should also increase the strength of the WFE (see Fawcett et al., 2013).

Thus, we created videos depicting a female perpetrator robbing a male victim. Depending on the experimental condition, the perpetrator held either a knife, a filled plastic water bottle, or a plunger. Both the perpetrator and the victim were White and in their late 20s. The videos were approximately 50 s long and contained no cuts or camera movements. They had a resolution of 1920 × 1080 pixels at 25 frames per second and subtended 47.5° horizontally and 27.8° vertically.

**Fig. 1:**
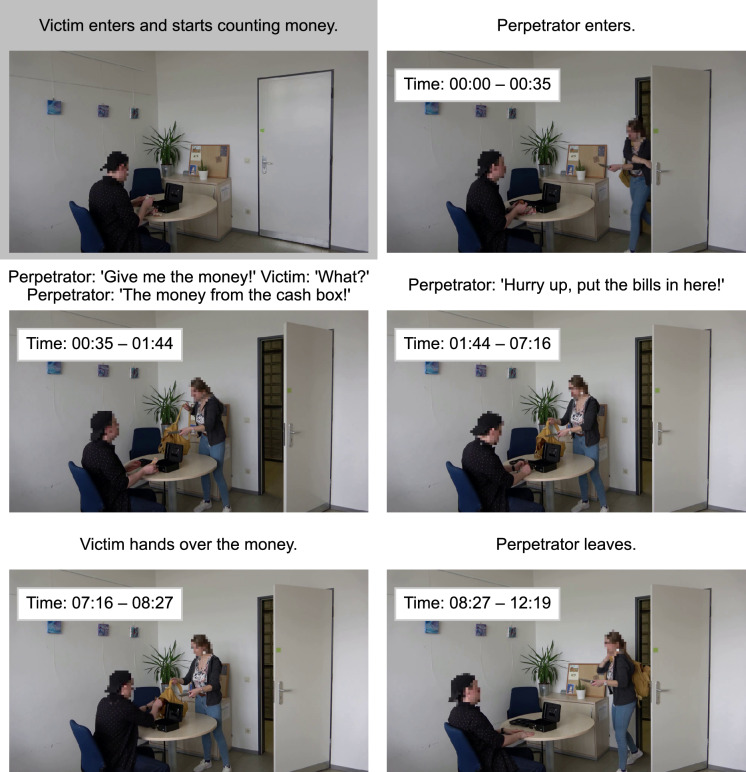
Screenshots illustrating key interactions from the video stimuli. The panel titles describe the actions performed and the words spoken (translated from German to English) during the various sections of the videos. The timestamps within the screenshots indicate the time in seconds during which the respective actions took place, relative to the beginning of the sequence for which eye movements were analyzed (i.e., the time during which the perpetrator was visible). The example frames are taken from the knife condition, but the timestamps are aggregated across object conditions. The panel at the top left (gray background) illustrates the actions taking place before the analyzed sequence. Faces were pixelated for privacy protection; note that this was not the case in the experimental stimuli

Figure 1 illustrates key sections of the videos with example frames and detailed timestamps. The videos started with a shot of a room containing a table, two chairs, and a cabinet. After a short while, the victim entered the room holding a cash box. He sat down at the table, opened the box, and started counting the money inside. Then, the perpetrator entered, carrying the critical object and a backpack. She demanded that the victim hand over the money. When the victim did not immediately comply, the perpetrator urged him to hurry. After the victim had put the money in the backpack, the perpetrator left, and the videos ended. The victim remained calm throughout the video. The perpetrator was visible for about 12 s (from second 34 to second 46). During this period, the critical object was also constantly in view.

### Apparatus and procedure

The stimuli were presented on a 24.5-in. monitor with a resolution of 1920 × 1080 pixels and a refresh rate of 144 Hz. Binocular eye movements were recorded using an SR Research EyeLink 1000 Plus Tower Mount, which includes a chin-and-forehead rest to minimize participants’ head movements. The viewing distance was 62 cm. The soundtrack of the videos was played through loudspeakers.

Participants were instructed to pay close attention to the content of the video. They were not informed that their memory for certain aspects of the video would be tested afterward. Before stimulus presentation, a nine-point calibration and validation of the eye tracker was performed. Moreover, the following fixation check was implemented: A fixation cross was presented at the center of the screen for 500 ms. The video was displayed only if the participant’s gaze remained within an area of 0.6° × 0.6° around the cross for at least 200 ms.

Immediately after participants had watched the video, they filled out a questionnaire regarding the appearance of the perpetrator. The questions pertained both to the perpetrator’s clothing (e.g., jacket, shirt, and pants) and to her physical characteristics (e.g., age, height, and hair style). There were both questions with predefined response categories (e.g., “Was the perpetrator wearing any headgear?” with the alternatives “yes” and “no”) and open-ended questions (e.g., “What type of headgear was it?”). When creating the stimulus material, care was taken to ensure that there were many details about the perpetrator’s appearance that subjects could potentially report. For example, the shirt of the perpetrator had both a complex pattern and a lettering, and she wore multiple accessories. Both the fact that we asked participants to recall details of the perpetrator’s appearance rather than to identify her in a line-up, and the fact that memory was tested immediately after stimulus presentation should also increase the strength of the memory-related WFE (see Fawcett et al., 2013). In addition to these questions probing participants’ memory, the questionnaire included a few rating items about the threat and unusualness of the critical object (see Experiment 3 in Körner et al., 2023, for details).

### Data analysis

The data were analyzed using SR Research Data Viewer, Python, and R (see Appendix 1 for software details). Eye movements were analyzed only for the sequence during which the perpetrator was visible. For each participant, only the data from the eye with the lower average error during the eye-tracker validation were analyzed (cf. Hooge et al., 2019). The empirically determined quality of the eye-tracking data was high, with a high accuracy (average validation error: *M* = 0.32°, SD = 0.07), high precision (root-mean-square sample-to-sample deviation: *M* = 0.0191°, SD = 0.0032), and low data loss (missing samples: *M* = 1.70%, SD = 3.36).

### Dynamic regions of interest

**Fig. 2:**
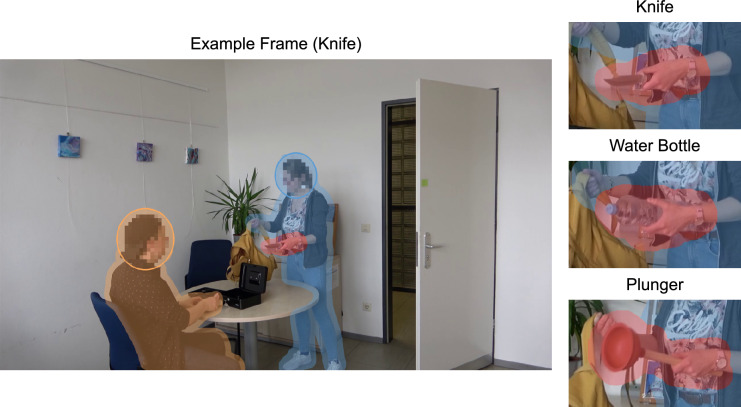
Examples of the stimulus material with superimposed regions of interest. Left panel: sample frame from the knife condition. Right panels: close-ups on the different critical objects, i.e., the knife, water bottle, and plunger. Transparent overlays mark the regions of interest for the perpetrator (blue), the critical object (red), and the victim (orange). The blue and the orange ellipses mark the borders of the region of interest for the head of the perpetrator and the victim, respectively. Faces were pixelated for privacy protection; note that this was not the case in the experimental stimuli

To analyze the allocation of attention and gaze, dynamic regions of interest (ROIs) were defined around the perpetrator, the victim, and the critical object (see Fig. 2). Additional ROIs were created for the heads of the perpetrator and the victim to allow for a more fine-grained analysis. For our main analyses, viewing times on the people were determined by adding the viewing times on their head and their body. To account for inaccuracies in the eye-tracking hardware, a margin of approximately 0.5° was added to each ROI (see Orquin et al., 2016).

All ROIs were generated automatically. Specifically, image-segmentation machine-learning algorithms were used to detect in each video frame the image regions belonging to the critical object or to one of the depicted people. The ROIs for the critical objects were created using the unsupervised semantic-segmentation framework *STEGO* (Hamilton et al., 2022) with a *DINO* backbone (Caron et al., 2021) and a refinement using a fully-connected conditional random field (Krähenbühl & Koltun, 2011). The ROIs for the perpetrator and the victim were created using the neural-network module *PointRend* (Kirillov et al., 2020) with a *ResNet-101* backbone (He et al., 2016). To create the ROIs for the heads of the perpetrator and the victim, ellipses were fitted to the top regions of the ROIs covering the entire body. This method was chosen because common face-detection algorithms failed to reliably detect the victim’s face.

Generating the ROIs automatically yielded ROIs that closely matched the actual targets. This close fit was especially important for the critical objects. As illustrated in Fig. 2, the individual critical objects, and consequently their corresponding ROIs, differed in size to some extent. Specifically, the ROI was largest for the plunger and smallest for the knife. Larger objects are generally more likely to be fixated than smaller ones (e.g., Nuthmann et al., 2020). In the context of the WFE, the assumed attentional effects of the critical objects are based on the different meanings of the objects and therefore should persist across various visual characteristics. This independence of the WFE from visual characteristics is supported to some extent by studies reporting similar memory effects for weapons of an unusual type or appearance compared to more conventional weapons (McRae et al., 2014; Pickel et al., 2006). To test whether the different ROI sizes affected the results, we also analyzed the data for critical-object ROIs which were equalized in their average size (see Appendix 2). However, using size-adjusted ROIs did not substantially alter the results, suggesting that the size differences were not pivotal to our findings.

Some of the ROIs overlapped. Specifically, the critical object ROI overlapped with the perpetrator ROI. There was virtually no overlap between the ROI for the critical object and the victim ROI, and the perpetrator ROI also did not overlap with the victim ROI. Naturally, the head ROIs for each person overlapped with their body ROIs. Due to these overlaps, gaze samples could fall into more than one ROI. In these cases, gaze samples were still assigned to a single ROI only. Specifically, gaze samples were assigned primarily to the critical object, and then to the person ROIs. Within each person ROI, the head ROI was given priority over the body ROI.

### Memory score

Subject responses were scored by a rater who was unaware of the underlying hypotheses. Each response was coded as either correct or incorrect, depending on whether it matched descriptions in an answer key. For the open-ended questions, each new piece of information provided by a given subject was counted as one detail. For example, if a subject reported that the perpetrator had worn a black belt, this would be counted as one correct detail (the perpetrator did wear a belt) and one incorrect detail (the belt was brown, not black). A subset of 90 subject responses was evaluated by a second, independent rater (cf. Pickel & Sneyd, 2018). Inter-rater reliability was high for both correct (*r* = .98) and incorrect (*r* = .93) details. For the analyses, only data from the rater who scored all subject responses were used. We used the proportion of correct responses (i.e., the number of correct details divided by the total number of details, correct and incorrect) as a metric for memory performance (see Kocab & Sporer, 2016).

### Details on statistical analyses

To account for potential smooth-pursuit eye movements, ROI matching was based on individual gaze samples as opposed to average fixation locations (see Körner et al., 2023, for details). Gaze samples classified as belonging to saccades were excluded from the analyses. Planned contrasts were used to compare the experimental groups. We complement results from null-hypothesis significance testing with Bayes factors (Jeffreys, 1935). While null-hypothesis significance testing provides researchers with a clear criterion on whether or not to accept the alternative hypothesis, Bayes factors also offer insights into how congruent the data are with the null hypothesis. Specifically, the Bayes factor *BF*_01_ is defined as the ratio of the probability for the observed data to have arisen under the null hypothesis to the probability for them to have arisen under the alternative hypothesis. If the null hypothesis and the alternative hypothesis explain the data equally well, *BF*_01_ is 1. In contrast, a value of *BF*_01_ = 5 would indicate that it is 5 times more likely for the data to have arisen under the null hypothesis than under the alternative hypothesis. Thus, the larger the value of *BF*_01_, the greater the support for the null hypothesis. Conversely, values below 1 indicate evidence in favor of the alternative hypothesis. We present the results using classifications proposed in the literature (Wagenmakers, Love, et al., 2018, Wagenmakers, Marsman, et al. 2018). Default priors (Morey et al., 2011; Rouder et al., 2009) were used.

## Results

### Total viewing time (general)

**Fig. 3:**
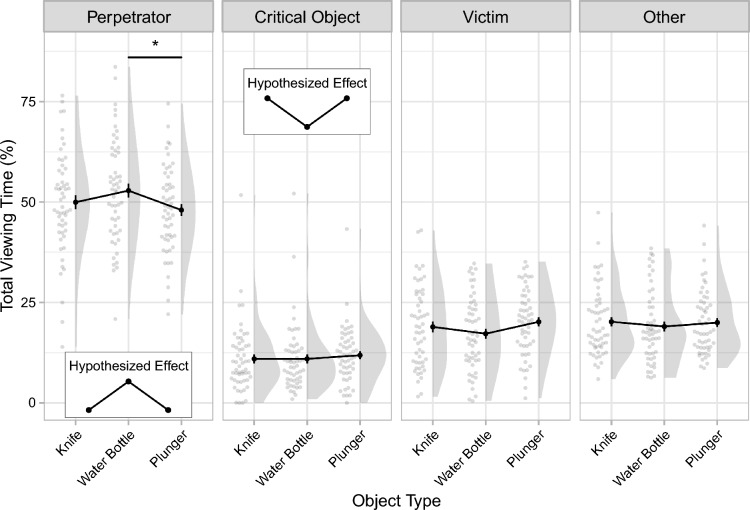
Total viewing times in Experiment 1. Relative total viewing times are shown as a function of object type for the different regions of interest (see panel titles). The black dots represent the means. Each of the smaller, gray dots represents data from an individual participant. The larger gray areas show the distribution of the data. The insets present the expected pattern of results for the perpetrator and the critical object, respectively. Note that the connecting lines between the discrete levels of object type are included only to facilitate comparisons between experimental conditions. Error bars are ± 1 SE. *p < .05

The total viewing time (TVT) is the summed duration of all gaze samples classified as fixations that landed within a given ROI. TVT was standardized as the percentage of the duration of the video segment during which the perpetrator was visible. Figure 3 and Table 1 show the relative TVT for the perpetrator, the critical object, the victim, and any area outside of these ROIs. Table 2 shows the results of the planned contrasts.

**Table 1:** Means and standard deviations for the total viewing times (%) in Experiment 1

Region of interest	Knife	Water bottle	Plunger
Perpetrator	49.94 (12.82)	52.83 (12.88)	48.00 (10.83)
Head	36.29 (14.06)	43.12 (13.52)	40.65 (12.57)
Body	13.65 (8.90)	9.71 (6.46)	7.35 (5.16)
Critical object	10.95 (8.42)	10.95 (8.47)	11.85 (7.01)
Victim	18.92 (9.98)	17.21 (9.23)	20.17 (8.29)
Head	7.57 (6.25)	9.19 (7.25)	8.44 (6.40)
Body	11.35 (8.24)	8.02 (6.11)	11.73 (7.78)
Other	20.19 (8.53)	19.01 (8.88)	19.98 (7.97)

Standard deviations are presented in parentheses. *N* = 162 (*n* = 54 for each condition)

**Table 2:** Results of the planned contrasts for the total viewing times in Experiment 1

Region of interest	Tails	*t*	*p*	*d*	*BF*_01_	Evidence for *H*_1_
*Perpetrator*						
Knife vs. Water Bottle	One	1.17	.123	0.22	1.55	−
Plunger vs. Water Bottle	One	2.11	.019	0.41	0.35	+
Knife vs. Plunger	Two	0.85	.395	0.16	3.54	− −
*Critical object*						
Knife vs. Water Bottle	One	< 0.01	.501	< 0.01	4.92	− −
Plunger vs. Water Bottle	One	0.60	.274	0.12	2.91	−
Knife vs. Plunger	Two	0.61	.544	0.12	4.16	− −
*Victim*						
Knife vs. Water Bottle	Two	0.92	.358	0.18	3.35	− −
Plunger vs. Water Bottle	Two	1.75	.083	0.34	1.25	○
Knife vs. Plunger	Two	0.71	.480	0.14	3.92	− −
*Other*						
Knife vs. Water Bottle	Two	0.71	.482	0.14	3.93	− −
Plunger vs. Water Bottle	Two	0.60	.550	0.12	4.18	− −
Knife vs. Plunger	Two	0.13	.895	0.03	4.87	− −

All *t*s are absolute values. *df* = 106 for all tests. Classifications for the evidence in favor of the alternative hypothesis *H*_1_ based on the Bayes Factor *BF*_01_: − − = moderate evidence against *H*_1_; − = weak evidence against *H*_1_; ○ = inconclusive evidence; and + = weak evidence in favor of *H*_1_

In all three experimental conditions, participants on average dedicated approximately half of their viewing time to the perpetrator. Contrary to predictions by current theories of the WFE, yet consistent with results by Körner et al. (2023), TVT on the perpetrator did not differ significantly between the knife and the water-bottle condition. In contrast, the presence of the plunger significantly reduced TVT on the perpetrator compared with the water-bottle condition. TVTs on the perpetrator did not differ significantly between the knife and the plunger condition.

On average, the various critical objects received only 11–12% of the overall viewing time. Neither the knife nor the plunger attracted significantly more gaze than the water bottle. TVTs on the knife and the plunger also did not differ significantly. Similarly, there were no significant differences between any of the experimental conditions in TVTs on the victim or on regions outside of the predefined ROIs.

### Total viewing time (heads versus bodies)

**Fig. 4:**
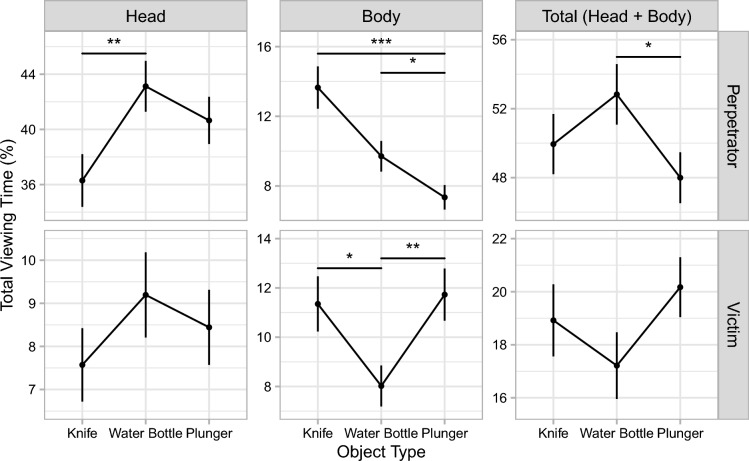
Total viewing times in Experiment 1 by body region. Mean relative total viewing times are shown as a function of object type for the different body regions (columns) of the perpetrator and the victim (rows). Note that the scales of the *y*-axes differ for the various regions of interest. Also note that the connecting lines between the discrete levels of object type are included only to facilitate comparisons between experimental conditions. Error bars are ± 1 SE. *p < .05. **p < .01. ***p < .001

A closer inspection of the viewing time on the depicted people revealed that object type affected TVT differently for the heads of the depicted people than for their bodies (see Fig. 4 and Tables 1 and 3). For the perpetrator, most fixations were directed toward her head in all object conditions, with her body receiving substantially shorter TVTs. In the knife condition, TVT on the perpetrator’s head was reduced compared with the water-bottle condition. Interestingly, this decrease in TVT on the head was offset by an increase in TVT on the body. Note that because current theories predict TVT on the perpetrator to be lower in the presence of a weapon compared to a neutral object, the clear increase in TVT on the perpetrator’s body in the knife condition was not significant in the respective one-tailed test.

**Table 3:** Results of the planned contrasts for total viewing times by body region in Experiment 1

Region of interest	Tails	*t*	*p*	*d*	*BF*_01_	Evidence for *H*_1_
*Perpetrator’s head*						
Knife vs. Water Bottle	One	2.57	.006	0.50	0.13	+ +
Plunger vs. Water Bottle	One	0.98	.163	0.19	1.93	−
Knife vs. Plunger	Two	1.70	.093	0.33	1.36	○
*Perpetrator’s body*						
Knife vs. Water Bottle	One	2.63	.995	0.51	16.77	− − −
Plunger vs. Water Bottle	One	2.10	.019	0.40	0.36	+
Knife vs. Plunger	Two	4.50	< .001	0.87	< 0.01	+ + + + +
*Victim’s head*						
Knife vs. Water Bottle	Two	1.25	.216	0.24	2.46	−
Plunger vs. Water Bottle	Two	0.57	.569	0.11	4.24	− −
Knife vs. Plunger	Two	0.72	.476	0.14	3.90	− −
*Victim’s body*						
Knife vs. Water Bottle	Two	2.39	.019	0.46	0.40	+
Plunger vs. Water Bottle	Two	2.76	.007	0.53	0.18	+ +
Knife vs. Plunger	Two	0.25	.806	0.05	4.78	− −

All *t*s are absolute values. *df* = 106 for all tests. Classifications for the evidence in favor of the alternative hypothesis *H*_1_ based on the Bayes Factor *BF*_01_: − − − = strong evidence against *H*_1_; − − = moderate evidence against *H*_1_; − = weak evidence against *H*_1_; ○ = inconclusive evidence; + = weak evidence in favor of *H*_1_; + + = moderate evidence in favor of *H*_1_; + + + + + = extreme evidence in favor of *H*_1_

In the plunger condition, TVTs on the perpetrator’s body were significantly lower than in both the water-bottle and the knife condition. TVTs on the perpetrator’s head did not significantly differ between the plunger condition and the other two object conditions. For the victim, TVTs on the body were significantly elevated in the knife and plunger conditions compared with the water-bottle condition. TVTs on the victim’s head were unaffected by object type.

### Cumulative time course (general)

**Fig. 5:**
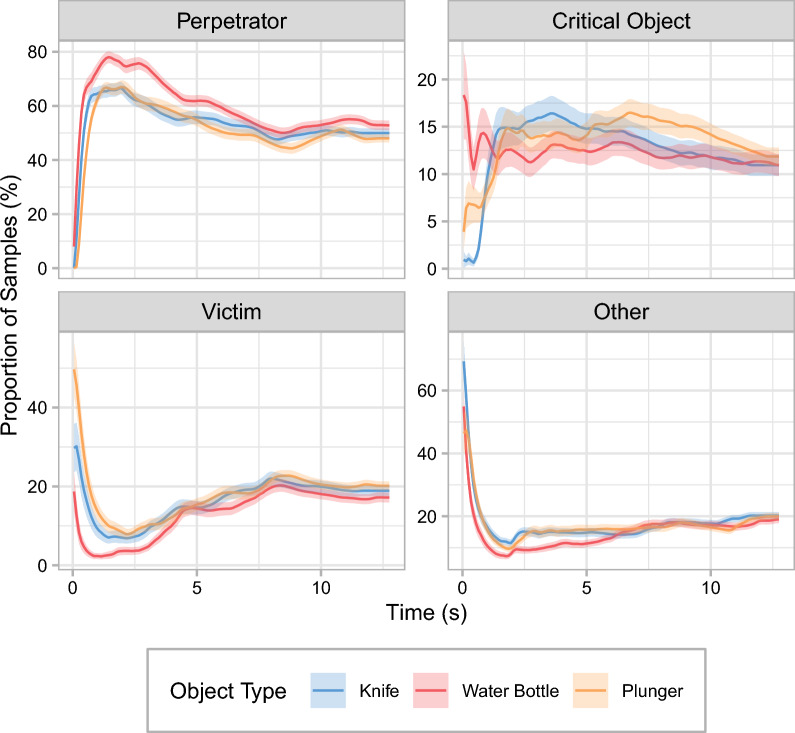
Cumulative time-course analysis for the gaze data in Experiment 1. The mean cumulative proportions of samples that landed within the different regions of interest (see panel titles) are shown as a function of time and object type. Individual gaze samples are grouped into 100-ms time bins. For each experimental condition, values at a given point in time add up to 100% across regions of interest. Note that the scales of the *y*-axes differ for the various regions of interest. Error ribbons are ± 1 SE

To more closely investigate participants’ allocation of attention, we also analyzed the proportion of gaze samples falling into the various ROIs over time. Figure 5 shows the cumulative relative proportion of samples for the different ROIs as a function of time and object type. Here, individual gaze samples were grouped into bins of width $\Delta t=100\,\text{ms}$. The interval ${T}_{k}=\left\{t|0<t\le k\Delta t\right\}$ accumulates the time up to the *k*th bin. The discrete cumulative time-course DCTC_*r*_ for a given ROI *r* within the time interval $(k-1, k]\Delta t$ then is

$${\text{DCTC}}_{r}\left(k\right)=\frac{\sum_{t\in {T}_{k}}{S}_{t,r}}{\sum_{t\in {T}_{k}}{S}_{t}},$$

where *S*_*t*_ are gaze samples at time *t*, and *S*_*t,r*_ are gaze samples that fell within the ROI *r* at time *t*. Finally, the cumulative time-course CTC_*r*_ for a given ROI *r* is

$${\text{CTC}}_{r}\left(t\right)={\text{DCTC}}_{r}\left(\lceil\frac{t}{\Delta t}\rceil\right)100.$$

Thus, each value along the *x*-axis marks the TVT for a given object condition and ROI up to that point in time. For example, the values at the 5-s mark show the TVT as reported above when only eye movements within the first 5 s after the perpetrator entered are considered. The values at the right end of Fig. 5 show the TVT for the entire duration that the perpetrator was visible and are therefore identical to the TVTs displayed in Fig. 3. Because each value of the cumulative time course corresponds to the TVT up to that point in time, this analysis offers insights into the effects that may have been observed for shorter exposure durations.

At the beginning of the scene, participants generally shifted their gaze toward the perpetrator. This is likely due to the fact that the victim and the rest of the scene were already present beforehand, whereas the perpetrator and the critical object she held were new and moving elements. Although this shift toward the perpetrator could be observed across all experimental conditions, it was less pronounced for the knife and plunger conditions compared with the water-bottle condition. For the knife and plunger conditions, most gaze samples were also allocated to the perpetrator as she entered, but a higher proportion of gaze samples remained on the victim and on regions outside of the defined ROIs compared with the water-bottle condition. The knife and the plunger themselves did not attract more gaze during this initial period than the water bottle. In fact, when considering only the very first second after the perpetrator had entered, both the knife and the plunger were looked at less than the water bottle.

In general, the differences between the experimental conditions diminished over time. Thus, object type affected attention allocation primarily at the beginning of the critical sequence, but these initial effects did not persist throughout the remainder of the sequence. As described above, when considering the total time during which the perpetrator was visible, the only significant effect remaining was a reduction in TVT on the perpetrator for the plunger condition compared with the water-bottle condition.

### Cumulative time course (heads versus bodies)

**Fig. 6:**
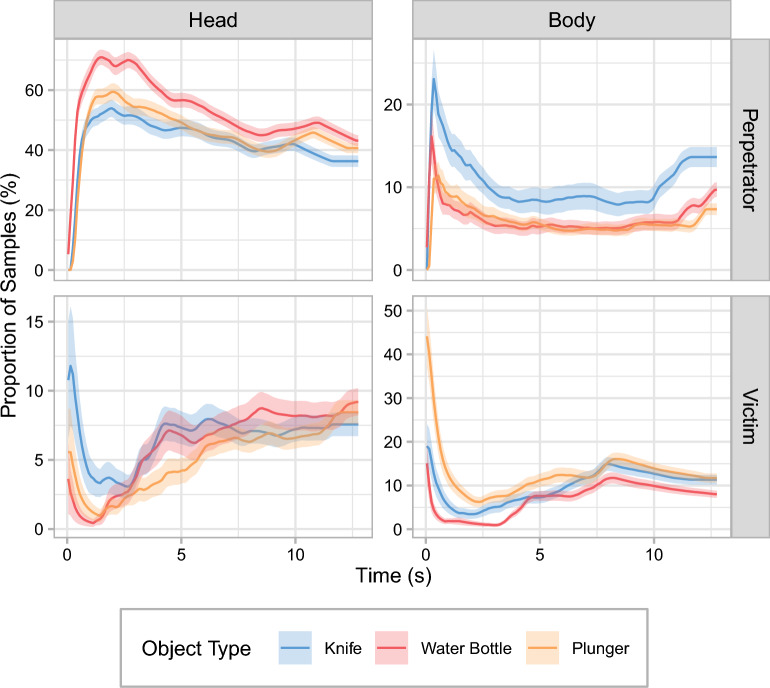
Cumulative time-course analysis by body region for the gaze data in Experiment 1. The mean cumulative proportions of samples that landed within the regions of interest for the head and the body (columns) of the perpetrator and the victim (rows) are shown as a function of time and object type. Individual gaze samples are grouped into 100-ms time bins. For each experimental condition, values at a given point in time add up to 100% across regions of interest (including the ones not presented in this figure). Note that the scales of the *y*-axes differ for the various regions of interest. Error ribbons are ± 1 SE

As with the TVT, we also examined the cumulative time course separately for the head and body ROIs of the perpetrator and the victim, respectively (see Fig. 6). For the perpetrator, the initial difference between the water-bottle condition on the one hand and the knife and plunger conditions on the other hand stems from a difference in viewing time on the perpetrator’s head rather than her body. Viewing time on the perpetrator’s body was elevated in the knife condition, and this difference to the other two object conditions remained nearly constant throughout the duration of the scene. For the victim, participants initially spent less time looking at both the victim’s head and his body in the water-bottle condition compared with both the knife and the plunger condition. While the decrease in viewing time on the victim’s head in the water-bottle condition was quickly compensated for, the difference in viewing time on the victim’s body persisted throughout the duration of the entire scene.

### Non-cumulative time course

**Fig. 7:**
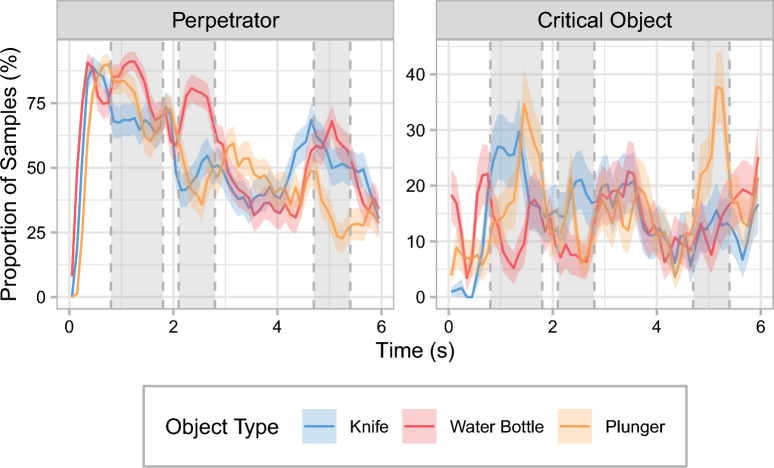
Non-cumulative time-course analysis for the gaze data in Experiment 1. The proportions of samples that landed within the regions of interest for the perpetrator and the critical object are shown as a function of time and object type. During the time periods highlighted by a gray background, either both the knife and the plunger (the first two intervals) or only the plunger (the third interval) received longer viewing times than the water bottle. These shifts toward the critical object each coincided with reduced viewing times on the perpetrator. Data are shown only for the first 6 s after the perpetrator entered. Individual gaze samples are grouped into 100-ms time bins. For each experimental condition, values at a given point in time add up to 100% across regions of interest (including the ones not presented in this figure). Note that the scales of the *y*-axes differ for the two regions of interest. Error ribbons are ± 1 SE

Finally, to more closely investigate the attentional allocation during the initial sequence of the scene, we also examined the non-cumulative time course for the first 6 s after the perpetrator entered for the ROIs of the perpetrator and the critical object (see Fig. 7). Here, individual gaze samples were again grouped into 100-ms time bins, but, in contrast to the cumulative analyses, each bin contains the data for that 100-ms interval only and not for all data up to that point in time.

The non-cumulative analysis reveals that while the knife and the plunger were looked at later than the water bottle, there were also time periods during which the knife and the plunger were focused on more than the water bottle (see the highlighted periods in Fig. 7). Specifically, there were peaks around the 1.5- and 2.5-s marks where both the knife and the plunger received roughly twice as much viewing time as the water bottle. Interestingly, these spikes in viewing time on the knife and the plunger each coincided with a decrease in time spent looking at the perpetrator. For the plunger, there was an additional third spike, which again paralleled a dip in viewing time for the perpetrator. These time periods each reflect moments in the videos in which the perpetrator is actively addressing the victim, urging him to hand over the money (see Fig. 1).

Thus, the non-cumulative analysis demonstrates that there were short periods of time for which the predicted attentional shift away from the perpetrator and toward the knife and plunger, respectively, could be observed. Our data suggest that the predicted attentional shift is more prominent in time periods which are (a) near the beginning of the scene and during which (b) the perpetrator assumes a more active role. However, as the cumulative analysis has shown, even in this initial sequence the knife and the plunger were overall not looked at for substantially longer than the water bottle. Instead, the spikes observed in the non-cumulative analysis only compensated for previous instances where the water bottle received a larger proportion of gaze samples compared with the knife and the plunger.

### Memory accuracy

**Fig. 8:**
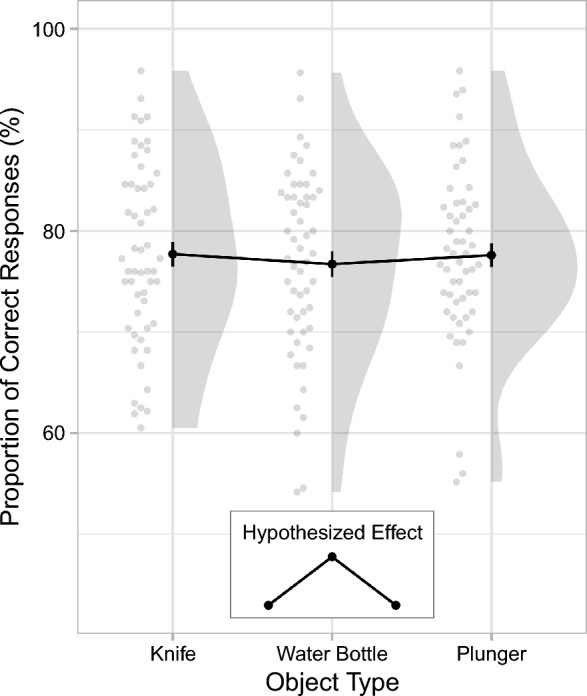
Memory accuracy in Experiment 1. Proportions of correct responses are shown as a function of object type. The black dots represent the means. Each of the smaller, gray dots represent data from an individual participant. The larger gray areas show the distribution of the data. The inset presents the expected pattern of results. Note that the connecting lines between the discrete levels of object type are included only to facilitate comparisons between experimental conditions. Error bars are ± 1 SE

Figure 8 and Table 4 show the effects of object type on memory accuracy. Memory performance was highly similar in all three experimental conditions. Contrary to expectations derived from previous WFE results, memory accuracy was not reduced by the presence of a knife (*M* = 77.71%, SD = 8.97) compared to the water bottle (*M* = 76.71%, SD = 9.17). Similarly, there was no decrease in memory performance in the plunger condition (*M* = 77.60%, SD = 8.57) compared to the water-bottle condition. Thus, there was no classic WFE, and, contrary to predictions by the unusual-item hypothesis, the unusual object also did not impair memory for the perpetrator’s appearance. There was no significant difference in memory performance between the knife and the plunger condition either.

**Table 4:** Results of the planned contrasts for the memory accuracy in Experiment 1

Comparison	Tails	*t*	*p*	*d*	*BF*_01_	Evidence for *H*_1_
Knife vs. Water Bottle	One	0.57	.716	0.11	7.19	− −
Plunger vs. Water Bottle	One	0.52	.698	0.10	6.97	− −
Knife vs. Plunger	Two	0.06	.949	0.01	4.90	− −

All *t*s are absolute values. *df* = 106 for all tests. Classifications for the evidence in favor of the alternative hypothesis *H*_1_ based on the Bayes Factor *BF*_01_: − − = moderate evidence against *H*_1_

## Discussion

Weapons and non-threatening unusual objects have long been theorized to have similar effects on attention and memory (E. F. Loftus et al., 1987). Previous research on the WFE in general and on the unusual-item hypothesis in particular has focused heavily on investigating memory effects, whereas attentional aspects have not been studied as extensively. Here, for the first time, we compared the attentional effects of a weapon and a non-threatening unusual object to those of a neutral control object using eye tracking and dynamic stimuli.

Weapon presence did not reduce viewing time on the perpetrator, and participants spent no more time looking at the weapon than at the neutral object. These findings are at odds with the attention-shift explanation inherent to current theories of the WFE. The results are, however, consistent with our previous findings regarding the attentional effects of weapon presence in dynamic scenes (Körner et al., 2023).

Similarly, the unusual object did not attract significantly more gaze than the other two objects. However, the perpetrator was looked at for a shorter duration when she carried an unusual object than when she held a neutral object. This reduction in viewing time on the perpetrator is partially consistent with the unusual-item hypothesis, although this hypothesis would predict a similar effect for the weapon, which was also unusual in the given (spatial) context. Viewing times on the victim and on regions outside of the predefined ROIs were not significantly affected by object type.

Thus, overall, attention allocation was similar across the various object conditions. However, a closer inspection revealed that object type did affect attention allocation on a more fine-grained level. For one, there was an interesting dissociation between viewing times on the perpetrator’s face and her body. Specifically, in the presence of a weapon, attention shifted from the perpetrator’s face toward her body. Thus, while weapon presence had no effect on *how long* observers looked at the perpetrator, it did affect *how* they looked at her.

Secondly, the effects of object type varied over time. Within the first few seconds after the perpetrator had entered, viewing time on the perpetrator was reduced for both the weapon condition and the unusual-object condition compared with the neutral-object condition. This initial reduction in viewing time on the perpetrator was driven exclusively by a decrease in viewing time on her face, with viewing time on the perpetrator’s body not being reduced for either the weapon condition or the unusual-object condition. In fact, viewing time on the perpetrator’s body was elevated rather than decreased throughout the duration of the scene for the weapon condition, but not the unusual-object condition.

Finding a decrease in attention paid to the perpetrator for both a weapon and a non-threatening unusual object is in line with predictions by the unusual-item hypothesis. Contrary to these predictions, however, the weapon and the unusual object themselves were not looked at for longer than the neutral object during this initial time period either. Instead, viewing times were increased for the victim and for other aspects of the scene besides the critical object and the depicted people. This shift toward the victim could indicate that observers wanted to assess how the victim would react to these unusual objects. As the videos progressed, these initial differences in attentional allocation did not persist, resulting in such effects being largely negligible in the overall viewing times (at least for the coarser ROIs covering the individuals as a whole).

The observed reduction in viewing time on the face of the person holding the weapon is also consistent with results by Biggs et al. (2013). In contrast, Körner et al. (2023) found no effect of weapon presence on viewing time on the perpetrator’s face. One possible explanation for this apparent inconsistency is a difference in exposure duration. In Körner et al. (2023), the perpetrator and the critical object were in view for a total of 32 s, whereas Biggs et al. (2013) presented isolated images for 5 s each, and the exposure duration in the current study was 12 s. A strong influence of exposure duration is further supported by the results of the time-course analyses. The differences in viewing time on the perpetrator’s face between the object conditions were most prominent during the first few seconds of the scene, with viewing times converging over time. In line with predictions by current theories, these initial attentional shifts away from the perpetrator were accompanied by a temporary focus on the weapon and the unusual object, respectively, although there were other time periods during which the neutral object received the most attention.

These results suggest that an initial decrease in attention paid to the perpetrator’s face may be reflected in overall viewing times for short exposure durations only. The idea that an attentional shift away from the perpetrator’s face is more prominent for shorter exposure durations is also consistent with memory results by Erickson et al. (2024), who found that weapon presence impaired participants’ ability to construct recognizable facial composites for an encoding duration of 10 s, but not for 30 s. Our results suggest that the differences in viewing time on the body of the perpetrator are less time-course dependent. The increase in viewing time on the perpetrator’s body due to the presence of a weapon remained approximately constant throughout the duration of the scene, indicating that this attentional shift may be a more stable and long-lasting phenomenon.

There were no effects of object type on memory performance: Participants recalled the appearance of the perpetrator as accurately when she was holding a weapon or an unusual object as when she was holding a non-threatening context-congruent object. This absence of a memory impairment is at odds with much of the previous research on the WFE (see Fawcett et al., 2013; Kocab & Sporer, 2016). Not finding a WFE is, however, consistent with the results of our previous study (Körner et al., 2023). Several other recent studies similarly failed to replicate the WFE, at least under certain conditions, and even found reversed effects, with weapon presence enhancing memory performance rather than impairing it (Harvey & Sekulla, 2021; Harvey et al., 2020; Mansour et al., 2019; Nyman et al., 2020).

## Experiment 2

To further investigate the inconsistencies between our memory results and previous research, Experiment 2 focused on the memory effects of weapons and unusual objects compared to a neutral object. We presented the same stimuli as in Experiment 1, but online and without eye tracking. We also investigated whether and how presenting stimuli with a soundtrack compared to no soundtrack modulates the memory-related WFE.

In the eye-tracking experiments of our previous study (Körner et al., 2023), we chose not to present the English soundtrack to our German participants. We argued that omitting the soundtrack should not matter since the WFE had previously been found both for presentations with sound (e.g., Carlson et al., 2017; Cutler et al., 1987; Pickel & Sneyd, 2018) and for presentations without sound (e.g., Carlson et al., 2016; Mitchell et al., 1998; Pickel, 1998, 1999). In an additional online experiment, where the soundtrack was included, we successfully replicated the memory results of the eye-tracking experiments (Körner et al., 2023).

Although consistent results indicate similar effects for presentations with and without sound, a systematic investigation of the impact of including versus omitting the soundtrack is still pending. While some studies have compared stimuli with and without soundtrack either between experiments (Kramer et al., 1990) or between subject groups (Beehr et al., 2004), these manipulations of sound were confounded with differences in other potentially relevant aspects of the stimuli, such as the presence versus absence of an assault or a shooting.

Based on current theories of the WFE, one could argue that the presentation of sound may modulate the effect. For one, omitting the soundtrack could enhance the WFE by increasing the ambiguity of the depicted situation. To increase ambiguity in this way, Pickel (1998) presented her stimuli without sound and deliberately avoided incorporating details into the material that would have conclusively established the depicted action as a robbery (e.g., the victim did not raise her hands in the air or react with fear). Our own videos similarly included no visual details that clearly identified the situation as a robbery. However, the words spoken by the perpetrator indicated that she was robbing the victim. If omitting the soundtrack leads observers to interpret the situation as a robbery in the weapon condition only, this would involve comparing an armed robbery on the one hand to interactions which are not interpreted as a crime and which do not involve a weapon on the other hand. Such a difference in interpretation could increase the classic WFE, although the potential effects are less clear for the non-threatening unusual object.

Conversely, one could reasonably contend that the WFE should be weaker, rather than stronger, when stimuli are presented without sound. For example, including the soundtrack could enhance participants’ immersion in the depicted situation, thereby increasing the impact of the weapon (cf. Beehr et al., 2004; Kramer et al., 1990). Finally, it is also possible that including a soundtrack does not, in fact, modulate the WFE.

## Methods

### Design, participants, stimuli, and questionnaire

The experiment had a 3 (object type: knife, water bottle, and plunger) × 2 (audio: with and without audio) between-subjects design. The sample consisted of *N* = 228 participants (166 women, 61 men, and 1 non-binary person). Subjects’ age ranged from 18 to 72 years of age (*M* = 27.2 years, SD = 11.2). The stimuli and questionnaire were the same as in Experiment 1.

### Procedure

The procedure was identical to that of Experiment 1, with the exception that Experiment 2 was conducted online and without the use of eye tracking. Participants were informed beforehand whether the video would have sound or not. In the conditions with audio, subjects were instructed to enable the sound on their device. The video could not be paused or replayed.

### Data analysis

As in Experiment 1, a subset of 90 participant responses were evaluated by a second rater. Inter-rater reliability was again high for both correct (*r* = .98) and incorrect (*r* = .90) details. Planned contrasts were used to compare the different object-type conditions within each audio condition.

## Results

### Memory accuracy

**Fig. 9:**
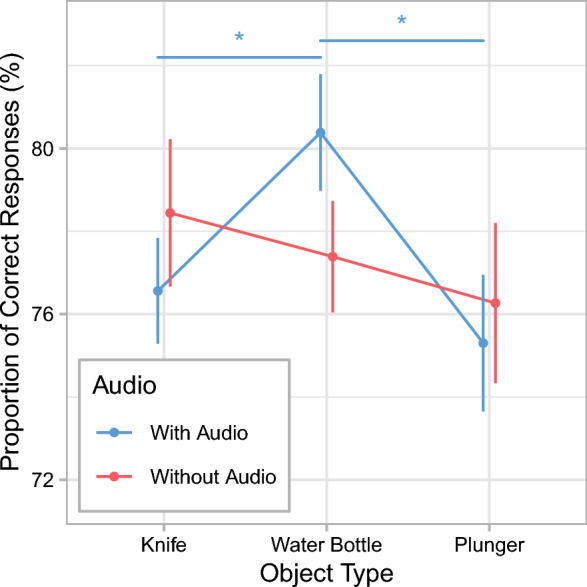
Memory accuracy in Experiment 2. Mean proportions of correct responses are shown as a function of object type and audio condition. Error bars are ± 1 SE. *p < .05.

Figure 9 and Tables 5 and 6 show the effects of object type and audio on the proportion of correct responses. For the conditions with audio, memory performance was best in the water-bottle condition. Memory for the perpetrator’s appearance was significantly worse when she carried a knife compared with a water bottle. Thus, there was a classic WFE. In line with predictions by the unusual-item hypothesis, memory accuracy was also significantly worse in the plunger condition than in the water-bottle condition. Memory performance did not differ significantly between the knife condition and the plunger condition. For the conditions without audio, memory performance was more similar for the various object types, with no significant differences between any of the object conditions. Thus, in contrast both to predictions by the unusual-item hypothesis and to the results in the conditions with sound, there was no memory impairment due to the presence of either a weapon or an unusual object when the stimuli were presented without sound.

**Table 5:** Memory accuracy (%) in Experiment 2

Condition	With audio		Without audio	
	*M*	SD	*M*	SD
Knife	76.56	7.87	78.44	10.97
Water Bottle	80.38	8.69	77.39	8.30
Plunger	75.30	10.17	76.27	11.91

*N* = 228 (*n* = 38 for each condition)

**Table 6:** Results of the planned contrasts for the memory accuracy in Experiment 2

Audio	Tails	*t*	*p*	*d*	*BF*_01_	Evidence for *H*_1_
*With audio*						
Knife vs. Water Bottle	One	2.01	.024	0.46	0.39	+
Plunger vs. Water Bottle	One	2.34	.011	0.54	0.21	+ +
Knife vs. Plunger	Two	0.60	.548	0.14	3.60	− −
*Without audio*						
Knife vs. Water Bottle	One	0.47	.681	0.11	5.78	− −
Plunger vs. Water Bottle	One	0.48	.318	0.11	2.85	−
Knife vs. Plunger	Two	0.83	.410	0.19	3.13	− −

All *t*s are absolute values. *df* = 74 for all tests. Classifications for the evidence in favor of the alternative hypothesis *H*_1_ based on the Bayes Factor *BF*_01_: − − = moderate evidence against *H*_1_; − = weak evidence against *H*_1_; + = weak evidence in favor of *H*_1_; + + = moderate evidence in favor of *H*_1_

### Ratings

Since the memory effects differed depending on the audio condition, we examined participants’ ratings of threat and unusualness (see Table 7). If the difference in memory effects between the audio conditions is due to a difference in perceived threat or unusualness, this should be reflected in these ratings. However, the pattern of results for the rating variables was the same for both audio conditions.

**Table 7:** Ratings of threat and unusualness in Experiment 2

Measure	With audio			Without audio		
	*n*	*M*	SD	*n*	*M*	SD
*Threat*						
Knife	38	7.47	2.52	37	7.05	2.32
Water bottle	35	1.14	1.88	33	1.76	2.54
Plunger	36	0.97	1.70	36	1.39	1.92
*Self-reported arousal*						
Knife	38	1.74	1.27	38	1.42	1.20
Water bottle	38	1.13	0.99	38	0.74	0.69
Plunger	38	0.95	1.16	38	1.00	0.74
*General unusualness*						
Knife	37	4.05	3.39	36	3.67	2.80
Water bottle	35	1.97	3.56	32	1.25	2.45
Plunger	37	6.22	3.60	36	6.03	3.33
*Situational unusualness*						
Knife	38	3.82	3.33	35	3.94	3.28
Water Bottle	35	8.71	2.61	32	8.78	1.90
Plunger	35	9.80	0.53	36	9.69	0.67

Threat as well as general and situational unusualness were rated on a scale from 0 to 10. Self-reported arousal was rated on a scale from 0 to 5

As expected, the knife was rated to be more threatening than both the water bottle and the plunger (all *p*s < .001, one-tailed tests). Similarly, self-reported arousal was significantly higher in the knife condition than in the other two object conditions (all *p*s < .035, one-tailed tests). Nevertheless, self-reported arousal, which was rated on a scale from 0 to 5, was still quite low in the knife conditions both with audio (*M* = 1.74, SD = 1.27) and without audio (*M* = 1.42, SD = 1.20). Ratings for threat and self-reported arousal did not differ significantly between the plunger and the water-bottle conditions (all *p*s > .11, two-tailed tests).

In line with our expectations, the general unusualness of the water bottle was rated lower compared with both the knife and the plunger (all *p*s < .007, one-tailed tests). The plunger was rated to be more unusual in general than the knife (all *p*s < .010, two-tailed tests). For the situational unusualness (i.e., the unusualness in the context of the video), a different pattern emerged. Here, the knife was judged to be the least unusual in the given situation (all *p*s < .001, two-tailed tests). The plunger was deemed more situationally unusual than the water bottle (all *p*s < .019, two-tailed tests).

## Discussion

In this online experiment, we investigated the memory effects of a weapon and a non-threatening unusual object compared to a neutral control object using the same stimuli as in Experiment 1. Moreover, we examined the effects of presenting stimuli either with or without sound. The effects of object type differed depending on whether the stimuli were presented with or without sound. When the videos included a soundtrack, memory was poorer in the presence of either a weapon or an unusual object compared with the neutral object. Thus, the results for the conditions with sound matched predictions by the unusual-item hypothesis as an explanation for the WFE. In contrast, object type had no significant effects on memory when the videos were presented without sound.

The fact that we found a WFE with sound, but no effects without sound is surprising. Previous research has shown that the WFE can occur both when stimuli are presented with sound (e.g., Carlson et al., 2017; Cutler et al., 1987) and when they are presented without sound (e.g., Carlson et al., 2016; Pickel, 1998). In our previous study (Körner et al., 2023), we found no memory-related WFE, and this result was consistent across experiments with and without sound. Importantly, the eye-tracking results of the current Experiment 1 are consistent with the findings of our previous study, demonstrating that these previous eye-tracking results are not limited to presentations without sound. Finally, finding a WFE in the conditions with sound of Experiment 2 is at odds with the memory results of Experiment 1. In Experiment 1, the same videos were also presented with sound, but no memory effects were observed for either the weapon or the unusual object.

It is unclear why the presence of sound seems to have modulated the WFE in our Experiment 2. Self-reported arousal was low in all conditions, although slightly higher in the presence of a weapon than for the other two objects, regardless of sound condition. This generally low level of self-reported arousal contradicts the assumption that including the soundtrack meaningfully increased participants’ emotional involvement in the videos. Moreover, the unusual object also impaired memory relative to the neutral-object condition, even though self-reported arousal was similar in both conditions.

Differing interpretations of the scene depending on the sound condition are also unlikely to explain our findings. Firstly, if participants in the non-weapon conditions interpreted the scene as a crime only when the videos were presented with sound, the WFE would be expected to be *weaker* in the conditions with sound, not stronger (see the reasoning above). Secondly, situational-unusualness ratings, which should depend on the interpretation of the scene, were highly consistent between the conditions with and without sound. However, it should be noted that the questionnaire about the woman’s appearance, which subjects completed prior to the ratings, unambiguously referred to the situation as a robbery and the woman as the perpetrator, which may have retroactively influenced participants’ interpretation of the scene.

## General discussion

Previous research has found that weapons and non-threatening unusual objects can impair memory to a similar extent (e.g., Mitchell et al., 1998; Pickel, 1998). The unusual-item hypothesis attributes this memory effect to an attentional shift from the perpetrator toward the objects themselves. The primary aim of the current study was to directly test this assumption by comparing the attentional effects of weapons and non-threatening unusual objects under naturalistic viewing conditions. In our main Experiment 1, we tracked participants’ eye movements while they watched a video of a staged robbery. Depending on the experimental condition, the perpetrator held either a weapon, an unusual object, or a neutral control object. Our results show that the allocation of attention between the weapon and the unusual object was more similar than between either object and the neutral object. However, the attentional effects of a weapon also differed substantially from those of a non-threatening unusual object in some respects.

The similarities between the weapon and the non-threatening unusual object were most prominent during the first few seconds of the scene. Results from the cumulative time-course analyses in Experiment 1 show that the perpetrator initially drew most attention when she held a neutral object, and viewing times on the actual weapon and the unusual object were initially lower than for the neutral object. For both the weapon and the unusual object, attention seemed to linger longer on the victim and on other aspects of the scene. In particular, viewing times on the victim’s body were increased compared with the neutral-object condition. This shift toward the victim’s body remained present throughout the duration of the entire scene, whereas many of the other initial differences did not persist even for the comparatively short exposure duration of 12 s used in the current study (see the discussion of Experiment 1 for details).

In contrast, the weapon and the non-threatening unusual object had different effects on overall viewing times on the perpetrator. In the weapon condition, the initially reduced viewing time on the perpetrator relative to the neutral-object condition was only temporary, whereas this reduction persisted until the end of the scene in the unusual-object condition. Moreover, observers focused on different aspects of the perpetrator depending on the type of unusual object. For the non-threatening unusual object, the reduced viewing time on the perpetrator relative to the neutral-object condition was primarily due to a reduced viewing time on her body. In contrast, the presence of a weapon led to an increase, rather than a reduction, in viewing time on the perpetrator’s body, but here observers paid less attention to her face.

The observed similarities between the weapon and the non-threatening unusual object support the idea that unusualness is a key feature influencing the effects weapons have on attention, whereas the clear differences in results indicate that other factors besides unusualness are also at play. The most natural explanation seems to be that the threat posed by weapons likewise affects observers’ allocation of attention (cf. Easterbrook, 1959; ﻿E. F. Loftus et al., 1987). However, it is important to consider that, despite the differences highlighted above, the overall distribution of viewing times was very similar across all three object conditions. This similarity suggests that attentional allocation was primarily driven by the interaction of the depicted people rather than by the specific object held by the perpetrator.

Results from the current study are consistent with our previous findings (Körner et al., 2023) on the attentional effects of weapons under naturalistic viewing conditions. Most notably, all critical objects on average received only 11–12% of the overall viewing time. These comparatively short viewing times are inconsistent with the notion of an extensive attentional focus on weapons or unusual objects. In contrast, participants in all object conditions dedicated about half of their viewing time to the perpetrator. The fact that, in the presence of a weapon, observers shifted their attention toward the bodies of the depicted people can be interpreted as a heightened focus on the interaction of the people, which would also be consistent with the time-course results reported by Körner et al. (2023). Interestingly, the shift toward the body of the perpetrator was not observed in the unusual-object condition, which suggests that the threat posed by the weapon, rather than its unusualness, is responsible for this effect. An explanation for the focus on the perpetrator’s body could be that observers were looking for signs that the perpetrator was about to use the knife, such as a movement of the arm.

Our eye-tracking results contrast some findings from the broader literature on context-incongruent objects (e.g., Henderson et al., 1999; G. R. Loftus & Mackworth, 1978). Specifically, neither the non-threatening unusual object nor the weapon, which was also incongruent to the given context, attracted more attention than a context-congruent control. This effect was also absent at the beginning of the scene. The context-incongruent objects were not looked at earlier than the congruent object either. If anything, there was an inverse effect, with the congruent object being looked at earlier than the incongruent objects (see Figs. 5 and 7).

This apparent inconsistency with previous findings is probably due to a number of methodological differences. Firstly, we used video stimuli, and the critical objects themselves as well as the depicted people and the objects they touched were moving. Secondly, the fact that our stimuli included people deviates from the common practice of using images without individuals in studies on object–scene (in)congruencies. Finally, in contrast to studies using static images, our stimulus material did not feature a defined scene onset. Instead, eye movements were analyzed for the duration for which the perpetrator and the critical object were visible. At the time the perpetrator entered the room, the observer had already processed the victim and the rest of the scene. Clearly, future research is needed to determine how these methodological aspects influence the attentional effects of context-incongruent objects.

A second aim of the current study was to compare the *memory* effects of weapons and non-threatening unusual objects. The unusual-item hypothesis proposes that, as a consequence of an assumed attentional shift from the perpetrator toward unusual objects, encoding is impaired for details of the perpetrator’s appearance. We found mixed results in regard to this memory-related WFE. In Experiment 1, the different critical objects did not affect memory performance. This seems generally consistent with our eye-tracking results, where we found similar viewing times for the perpetrator across all object conditions. Although the presence of a non-threatening unusual object was associated with a reduced overall viewing time on the perpetrator, this did not translate into a memory impairment. In Experiment 2, however, we did observe a memory impairment for both the weapon and the unusual-object condition relative to the neutral-object condition when the videos were presented with sound, but found no effects of object type when the videos were presented without sound. Since we used the same videos in both experiments and included the soundtrack in Experiment 1, the memory findings of our two experiments are contradictory.

The only difference in experimental design between Experiment 1 and the conditions with sound in Experiment 2 was that Experiment 2 was an online study, whereas Experiment 1 included eye tracking and was conducted in the laboratory. The WFE has been investigated in various experimental settings, including online studies (e.g., Carlson et al., 2017; Erickson et al., 2014), group sessions (e.g., Kramer et al., 1990; Pickel, 2009), individual laboratory sessions (e.g., Harvey & Sekulla, 2021; Mansour et al., 2019), and live-simulation studies (e.g., Maass & Köhnken, 1989). How these different settings may affect the WFE has not been studied extensively, although Pickel and Sneyd (2018) found no significant differences between data obtained online versus in group laboratory sessions.

Another potentially relevant difference between our Experiments 1 and 2 is the sample composition. Within online studies, it is easier to recruit a more diverse sample, whereas laboratory research often relies more heavily on student participants. This difference is reflected in the sample compositions of the laboratory Experiment 1 and the online Experiment 2. For example, the average age was higher and the age range wider in Experiment 2 than in Experiment 1. Future research is needed to assess whether and to what extent the experimental setting as well as different sample characteristics modulate the WFE.

Alternatively, it may also be the case that the inconsistency in results is simply due to chance. Considering the data from all conditions in both experiments, it seems more likely that, in the conditions with sound of Experiment 2, memory was incidentally elevated in the neutral-object condition, rather than being impaired in the presence of the weapon or the unusual object. Average memory accuracy was above 80% in this specific condition, whereas it ranged from 75 to 78% in all other conditions. The idea that the slightly higher memory performance in this condition may be a statistical fluke is supported to some extent by the fact that, for the comparison between the weapon and the neutral object, the corresponding Bayes factor was below the conventional threshold for the evidence to be considered more than anecdotal. For the unusual object, however, the Bayes factor indicated moderate evidence in favor of a true difference from the neutral object. Still, compared with this evidence in favor of a memory-related WFE obtained in the conditions with sound of Experiment 2, the results of Experiment 1 provided stronger evidence *against* a memory impairment caused by either a weapon or an unusual object.

It should be noted that because we failed to find a memory-related WFE in Experiment 1, it is unclear to what extent the eye-tracking results from this experiment generalize to a sample that does show the classic WFE. However, there is reason to think that the inconsistency between our data and previous studies providing evidence in favor of the attention-shift hypothesis is more likely to be due to the type of stimulus material used than to sample characteristics. When using slide shows in our previous study (Körner et al., 2023), we were able to replicate the eye-tracking results of the classic study by ﻿E. F. Loftus et al. (1987), even though we did not find a memory effect in that experiment either. Moreover, viewing behavior for dynamic scenes has been shown to be strongly influenced by bottom-up factors such as motion (e.g., Hutson et al., 2022; Itti, 2005; Loschky et al., 2015), which implies that viewing behavior is similar across observers. Thus, samples that do show a memory-related WFE may still fail to exhibit an attentional shift from the perpetrator to the weapon for dynamic scenes. Note, however, that we found significant differences in gaze behavior between our experimental conditions, suggesting that the tyranny of film was incomplete for our more naturalistic dynamic scenes (cf. Dorr et al., 2010; Levin et al., 2021). In any case, until evidence for the attentional shift predicted by current theories is provided for more naturalistic viewing conditions, the attention-shift explanation for the WFE should be considered tentative.

In the current study, we used stimulus material deliberately created to elicit a maximum memory effect based on known moderator variables of the WFE. For example, a knife was used as the weapon, the perpetrator was portrayed by a woman, and exposure duration and retention interval were within the ranges found to produce the strongest effects (see Fawcett et al., 2013; Kocab & Sporer, 2016). Therefore, the fact that we failed to find a memory effect both in Experiment 1 and in the no-sound conditions of Experiment 2 is striking. Even the memory effect we did observe in the conditions with sound of Experiment 2 was substantially smaller than what would be expected under these rather extreme circumstances.

In our previous study (Körner et al., 2023), we discussed cultural differences as a possible reason for not finding a memory-related WFE. Specifically, we argued that a difference in “gun culture” between the USA and Europe could be responsible for the fact that we failed to find a WFE despite using material for which large memory effects had been demonstrated in a US study (Pickel & Sneyd, 2018). However, cultural differences are unlikely to justify the absence of a WFE in Experiment 1 and the no-sound conditions of Experiment 2. Firstly, a knife was used as the weapon, for which cultural differences between the USA and Europe are presumably less pronounced than for firearms. Secondly, one would not necessarily expect the effects of non-threatening unusual objects to be modulated by cultural differences, provided the objects in question are in fact unusual in the given culture. In light of the present results, it is therefore unlikely that our repeated failures to replicate the memory-related WFE are due to cultural specificities.

## Conclusion

Our results show that gaze behavior was similar for a weapon and a non-threatening unusual object, supporting the idea that the unusualness of weapons in many contexts is a key factor influencing their effects on the allocation of attention. However, there were also differences between the effects of the weapon and the unusual object, which indicates that other features of weapons, such as their threatening nature, are also important. Contrary to predictions by current theories, neither the weapon nor the non-threatening unusual object drew attention away from the perpetrator for an extended period of time. While both objects reduced the time spent looking at the perpetrator initially, these effects were short-lived and had little impact on the overall viewing times. The present results corroborate our previous findings (Körner et al., 2023) suggesting that the attentional effects of weapons are more complex than the previously assumed shift away from the perpetrator toward the weapon. One interesting finding was that, in the presence of a weapon, observers shifted their attention from the perpetrator’s face to the body, possibly to assess whether the perpetrator was about to use the weapon.

## Figures and Tables

**Fig. 10:**
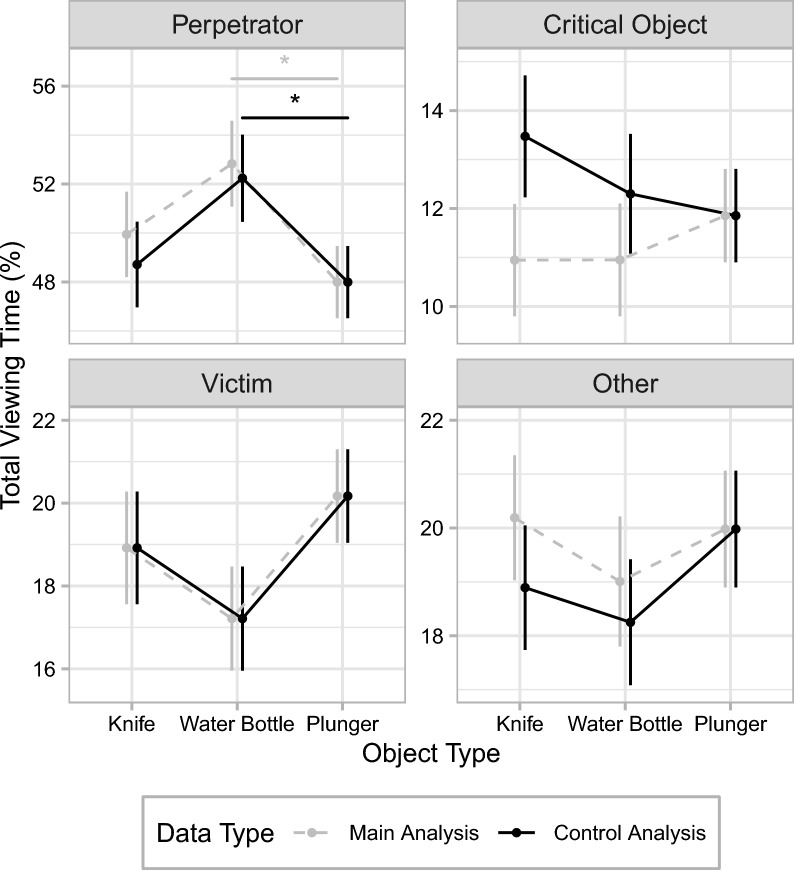
Total viewing times in Experiment 1 (control analysis versus main analysis). Mean relative total viewing times are shown as a function of object type and analysis method. Each panel presents the total viewing times for a different region of interest (see panel titles). Note that the scales of the *y*-axes differ for the various regions of interest. Also note that the connecting lines between the discrete levels of object type are included only to facilitate comparisons between experimental conditions. Error bars are ± 1 SE. *p < .05

## Data Availability

All data and analysis code are available in an OSF repository (https://osf.io/qk85u). The repository also contains the video stimuli used in the experiments, but to protect the privacy of the actors, the faces have been pixelated, and the soundtrack has been removed. The code used to generate the regions of interest for the critical objects can be found at: https://github.com/HannesMK/ROI-generation-STEGO. None of the experiments was preregistered.

## Appendix 1

### Software details

The eye-tracking experiment was implemented in MATLAB (Version R2019b) using the Psychophysics Toolbox extension (Brainard, 1997; Kleiner et al., 2007; Pelli, 1997), which incorporates the EyeLink Toolbox (Cornelissen et al., 2002). The online experiment was implemented in LimeSurvey (Version 2.23.3). All ROIs were generated in Python (Version 3.7.9) using the libraries *azureml-core* (Version 1.54.post1; Microsoft, 2023), *hydra-core* (Version 1.3.2; Yadan, 2023), *matplotlib* (Version 3.5.3; Hunter, 2007), *numpy* (Version 1.21.6; Harris et al., 2020), *omegaconf* (Version 2.3.0; Yadan, 2022), *opencv-python* (Version 4.8.1.78; Bradski, 2000), *pandas* (Version 1.3.5; McKinney, 2010), *pillow* (Version 9.5.0; Clark, 2023), *pixellib* (Version 0.7.1; Olafenwa, 2021), *pydensecrf* (Version 1.0rc3; Beyer, 2018), *pyhelpers* (Version 1.4.6; Fu, 2023), *pytorch-lightning* (Version 1.9.5; Lightning AI, 2023), *scikit-image* (Version 0.19.3; van der Walt et al., 2014), *scipy* (Version 1.7.3; Virtanen et al., 2020), *seaborn* (Version 0.12.2; Waskom, 2021), *tensorboard* (Version 2.11.2; Google, 2023), *torch* (Version 1.13.1; Paszke et al., 2019), *torchvision* (Version 0.14.1; TorchVision maintainers & contributors, 2022), *tqdm* (Version 4.66.1; tqdm developers, 2023), and *wget* (Version 3.2; Techtonik, 2015).

SR Research Data Viewer (Version 4.3.210) was used to process the raw eye-movement data. Both eye-movement data and behavioral data were then analyzed in R (Version 4.4.1) using the packages *BayesFactor* (Version 0.9.12–4.7; Morey & Rouder, 2024), *devEMF* (Version 4.4–2; Johnson, 2024), *effsize* (Version 0.8.1; Torchiano, 2020), *ggdist* (Version 3.3.2; Kay, 2024), *ggh4x* (Version 0.2.8; van den Brand, 2024), *ggplot2* (Version 3.5.1; Wickham, 2016), *ggpp* (Version 0.5.8–1; Aphalo, 2024), *ggpubr* (Version 0.6.0; Kassambara, 2023), *ggsignif* (Version 0.6.4; Ahlmann-Eltze & Patil, 2021), *huxtable* (Version 5.5.6; Hugh-Jones, 2024), *knitr* (Version 1.48; Xie, 2015), and *tidyr* (Version 1.3.1; Wickham et al., 2024).

## Appendix 2

### Control analysis

Current theories of the WFE predict an attentional shift away from the perpetrator and toward the weapon. In our main analysis, we failed to find this predicted effect on viewing times both for the perpetrator and for the critical object. However, the ROI sizes for the critical objects used for this main analysis differed to some extent, which may have influenced the results. The goal of this control analysis is to check whether our failure to find the predicted attentional WFE could be attributed to this difference in ROI size.

For the main analysis, the ROI was smallest for the knife and largest for the plunger. Larger objects are generally more likely to be fixated than smaller objects (e.g., Nuthmann et al., 2020). Thus, the predictions of current theories on the one hand and size-based considerations on the other hand are contradictory, with current theories predicting viewing times on the knife to be longest and size-based considerations predicting viewing times on the knife to be shortest. It is therefore important to investigate the influences that object ROI size has on the viewing times of Experiment 1. To maximize the chances of detecting a corresponding effect in the control analysis, we chose to equalize the average ROI sizes of all critical objects.

The size of the critical-object ROI affects not only viewing times on the critical object itself, but also viewing times on the perpetrator, because (a) the ROIs for the critical object and the perpetrator overlap, and (b) the object ROI was given priority when assigning gaze samples to ROIs, meaning that larger object ROIs occlude more of the perpetrator ROI. Consequently, increasing the size of the object ROI generally leads to shorter viewing times for the perpetrator. To explore the extent to which the differing sizes of the critical-object ROIs affect the results, we evenly increased the size of the smaller ROIs in all directions for each frame up to the point where the average size across frames was approximately equal for the ROIs of the knife, water bottle, and plunger.

As mentioned above, this analysis method maximizes the chance to observe the predicted attentional shift away from the perpetrator and toward the weapon. Since the knife had the smallest ROI in the main analysis, its ROI size is inflated the most. This leads to more gaze samples being assigned to the knife and, consequently, to longer viewing times on the knife. Conversely, inflating the knife ROI covers up larger portions of the perpetrator ROI, thereby reducing viewing times on the perpetrator. Thus, the implemented adjustment in ROI size, if it has any effect at all, should generally result in viewing times that are more congruent with the predicted shift away from the perpetrator and toward the weapon.

The results of the control analysis are shown in Fig. 10 and in Tables 8 and 9. The differences in results between the size-adjusted control analysis and the main analysis were small. The largest change was observed for the knife, for which the average TVT increased by approximately 2.5 percentage points compared with the main analysis. The overall pattern of results remained the same for all ROIs. As with the main analysis, the only significant difference between the various object conditions was a reduced viewing time on the perpetrator in the plunger condition compared with the water-bottle condition. Thus, contrary to the predictions of current theories, the presence of a knife again did not significantly reduce viewing time on the perpetrator, and the knife was not looked at for significantly longer than the water bottle.

**Table 8:** Means and standard deviations for the total viewing times (%) in Experiment 1 (control analysis)

Region of interest	Knife	Water bottle	Plunger
Perpetrator	48.71 (12.83)	52.24 (13.08)	48.00 (10.83)
Critical object	13.47 (9.14)	12.30 (8.98)	11.85 (7.01)
Victim	18.92 (9.98)	17.21 (9.23)	20.17 (8.29)
Other	18.89 (8.49)	18.25 (8.59)	19.98 (7.97)

Standard deviations are presented in parentheses.* N* = 162 (*n* = 54 for each condition)

**Table 9:** Results of the planned contrasts for the total viewing times in Experiment 1 (control analysis)

Region of interest	Tails	*t*	*p*	*d*	*BF*_01_	Evidence for *H*_1_
*Perpetrator*						
Knife vs. Water Bottle	One	1.41	.080	0.27	1.11	○
Plunger vs. Water Bottle	One	1.84	.035	0.35	0.57	+
Knife vs. Plunger	Two	0.31	.754	0.06	4.69	− −
*Critical object*						
Knife vs. Water Bottle	One	0.67	.251	0.13	2.72	−
Plunger vs. Water Bottle	One	0.29	.613	0.06	6.01	− −
Knife vs. Plunger	Two	1.03	.304	0.20	3.05	− −
*Victim*						
Knife vs. Water Bottle	Two	0.92	.358	0.18	3.35	− −
Plunger vs. Water Bottle	Two	1.75	.083	0.34	1.25	○
Knife vs. Plunger	Two	0.71	.480	0.14	3.92	− −
*Other*						
Knife vs. Water Bottle	Two	0.39	.697	0.08	4.58	− −
Plunger vs. Water Bottle	Two	1.09	.280	0.21	2.90	−
Knife vs. Plunger	Two	0.69	.494	0.13	3.97	− −

All *t*s are absolute values. *df* = 106 for all tests. Classifications for the evidence in favor of the alternative hypothesis *H*_1_ based on the Bayes Factor *BF*_01_: − − = moderate evidence against *H*_1_; − = weak evidence against *H*_1_; ○ = inconclusive evidence; + = weak evidence in favor of *H*_1_

The results of this control analysis demonstrate that the attentional WFE predicted by current theories is not observed for the present data even when the most sensitive analysis method for detecting such an effect is chosen. The overall similarity in results compared to the main analysis suggests that the differences in ROI size between the critical objects were not pivotal to our findings. Thus, the fact that we failed to find the attentional WFE cannot be explained by these differences in object ROI size.

## Abbreviations

WFE: Weapon-focus effect
ROI: Region of interest
TVT: Total viewing time
